# Hepatic tuberculosis in human immunodeficiency virus co-infected adults: a case series of South African adults

**DOI:** 10.1186/s12879-017-2222-2

**Published:** 2017-02-01

**Authors:** Lilishia Gounder, Pravikrishnen Moodley, Paul K. Drain, Andrew J. Hickey, Mahomed-Yunus S. Moosa

**Affiliations:** 1 Department of Infectious Diseases, Nelson R. Mandela School of Medicine, University of KwaZulu-Natal, Durban, South Africa; 2 Department of Virology, National Health Laboratory Service, Inkosi Albert Luthuli Central Hospital, 800 Bellair Road, Durban, Mayville 4058 South Africa; 3 Department of Global Health, Medicine, and Epidemiology, University of Washington, Seattle, USA; 4 Department of Surgery, Massachusetts General Hospital, Harvard Medical School, Boston, USA; 5 University of Maryland School of Medicine, Baltimore, USA; 6 King Edward VIII Hospital, Durban, South Africa

**Keywords:** HIV, TB, Liver, Granuloma, South Africa

## Abstract

**Background:**

Although *Mycobacterium tuberculosis* (TB) infection may cause extrapulmonary disease in HIV-infected adults, HIV-associated hepatic TB has been poorly characterized. Our objective was to describe hepatic TB in HIV-infected adults.

**Methods:**

Retrospective study of patients diagnosed with hepatic TB from 2005–2012 at Infectious Diseases Clinic, King Edward VIII Hospital, Durban, South Africa.

**Results:**

Among twenty cases of histology-confirmed HIV-associated hepatic TB, median CD4 count was 47 cells/μl (inter-quartile range 27–107 cells/μl) and 75% (15/20) of patients had pre-existing pulmonary TB. The most frequent clinical finding was hepatomegaly (85%). Liver enzyme abnormalities included elevated alkaline phosphatase (median 456 u/L, inter-quartile range 322–1,043 u/L) and gamma-glutamyltransferase (median 422 u/L, inter-quartile range 235–736 u/L). Acid-fast bacilli were cultured from liver tissue in 30% (6/20) of patients; 25% (5/20) identified as TB. With standard anti-TB therapy, liver enzymes improved within six months in 92% (11/12) of patients. One year after diagnosis, twelve patients resolved clinically, two patients developed drug-resistant TB and six patients died.

**Conclusion:**

In our case series of HIV-infected patients, hepatic TB occurred in patients with severe immunosuppression, who presented with hepatomegaly and abnormal liver enzymes. More than half of patients had resolution of liver function by six months however the 12-month mortality remained high.

## Background

Mycobacterium tuberculosis (TB) and Human Immunodeficiency Virus (HIV) cause a high rate of morbidity and mortality worldwide [1]. Approximately 1.2 million HIV-infected people developed active TB infection worldwide in 2014, of which 74% were in sub-Saharan Africa [2]. In South Africa, more than 50% of TB cases have HIV co-infection with the risk being greater at lower CD4 cell counts [2, 3].

Although TB most commonly causes a pulmonary infection, patients with advanced immunosuppression are at greater risk of developing extrapulmonary infection [3]. One manifestation of extrapulmonary TB is hepatic TB, and the characteristics of HIV-associated hepatic TB have not been fully described. In the pre-HIV era a South African study reported a 9.2% prevalence of hepatic TB among inpatients with unexplained hepatomegaly [4].

Hepatic TB can occur as a localized primary infection or by dissemination of a pulmonary infection, known as miliary hepatic TB [5]. Local hepatic TB occurs when ingested TB bacilli cross the gastrointestinal mucosal barrier to reach the liver via the portal vein [6]. Disseminated disease via the hepatic artery is more common, and results in multiple small tubercles in the liver [6].

The clinical, laboratory and radiologic features of hepatic TB are non-specific [4, 7–12]. Some patients have liver enzyme abnormalities which may worsen during the first few months of anti-retroviral therapy (ART) [13]. Since liver enzyme abnormalities are varied and causes may include opportunistic infections, malignancy, HIV cholangiopathy, drug-induced liver injury, viral hepatitis, hepatic steatosis and immune reconstitution inflammatory syndrome (IRIS), the diagnosis of hepatic TB is challenging and requires a high index of suspicion [14, 15].

A better understanding of the clinical presentation, biochemical abnormalities, immunological characteristics, radiographic features, microbiologic findings, histopathologic findings, and response to treatment of hepatic tuberculosis will assist in a rational approach to the interpretation of liver enzyme abnormalities in the HIV-infected patient. We therefore sought to describe hepatic TB among HIV-infected patients receiving care at a tertiary level hospital in Durban, South Africa.

## Methods

### Data sources

We conducted a retrospective patient chart review at King Edward VIII Hospital, a tertiary level teaching hospital located in Durban, KwaZulu-Natal. We included all patients diagnosed with hepatic TB from 2005 to 2012 at the Infectious Diseases Clinic (IDC), a referral-based specialist service focused on the management of complex infectious diseases including HIV and TB.

Patients had a thorough clinical examination, laboratory testing, appropriate imaging, and a liver biopsy at the time of presentation, and as clinically appropriate. All patients were treated with standard anti-TB therapy following local treatment guidelines [16]. We reviewed all available chart data from initial presentation and follow-up visits. Patients’ South African identity numbers were used to search the national Department of Home Affairs records to determine survival after follow-up.

### Diagnosis of Hepatic TB

We included patients as cases in our study if there was evidence of TB as indicated by a histopathology report and/or a microbiology report and/or radiologic evidence of TB at an extra-hepatic site.

All tissue and sputum specimens were stained with Auramine O stain (Merck KGaA, Darmstadt, Germany) to detect acid-fast bacilli. Mycobacterial cultures were performed using the Bactec MGIT system (Becton Dickinson, New Jersey, USA). The one percent proportion method was used to determine susceptibility of mycobacterial isolates to isoniazid, rifampicin, streptomycin, kanamycin, and ofloxacin [17, 18].

All tissue specimens were processed and reported on by a routine clinical histopathology laboratory. Findings were interpreted in the context of clinical and other laboratory information. Tissue samples were routinely assessed for the characteristic histologic pattern consistent with TB. These include:

1. Granulomas - nodular reactions containing transformed macrophages with or without surrounding inflammatory cells such as lymphocytes, eosinophils and multinucleated giant cells [19, 20].
2. Granulomatous inflammation - a distinctive pattern of chronic inflammation characterized by aggregates of activated macrophages with scattered lymphocytes with or without caseation [20].

### Statistical analysis

Study data were collected and managed using Research Electronic Data Capture (REDCap™), an electronic data capture tool [21]. All patient variables were analyzed using Microsoft Excel 2007 (Microsoft Corporation, Washington, USA).

## Results

We identified twenty patients with HIV-associated hepatic TB (Table 1). Fourteen out of twenty patients (70%) were female, and median age was 34.5 years (range 22–58 years). All were HIV-infected and fifteen out of twenty patients (75%) were ART-naïve. At the time of liver biopsy, three out of twenty patients (15%) were on ART for a median of 111 days (range 14–235 days). The most frequent indication for liver biopsy was an unexplained liver function test abnormality, with or without hepatomegaly, in nineteen out of twenty patients (95%). One patient had a liver biopsy for isolated hepatomegaly.

**Table 1:** Patient characteristics and outcome

Pt	Gender	Clinical features			Laboratory markers at time of liver biopsy						Chest X-ray findings	Abdominal (and other) ultrasound findings	Duration of anti-TB therapy pre-liver biopsy (Days)	Duration of anti-TB therapy post-liver biopsy (Months)	Outcome at 1 year follow-up
		H	J	S	CD4 cells/μl	TBil μmol/l	ALT u/l	ALP u/l	GGT u/l	ALB g/l					
1	F	Y	N	N	43	15	49	2339	1268	17	Normal	ALN	7^a^	13	R
2	F	Y	Y	N	55	40	56	106	194	17	Normal	H, ALN	9	9	R
3	M	N	N	N	17	7	98	1035	275	23	Extensive paratracheal and hilar shadowing, active TB, nodular shadowing	Normal	140	5	D
4	F	Y	Y	Y	6	35	32	654	547	15	Consolidation with breakdown in RUZ, cavitation	Findings NR	50	7	R
5	M	N	N	Y	175	31	110	280	194	14	Large paratracheal lymph nodes	Findings NR	Post-biopsy	9	R
6	F	N	N	N	96	44	94	1110	994	16	Reticulonodular shadowing, paratracheal lymph nodes	Findings NR	6	NR	R
7	F	Y	N	N	56	3	35	1088	873	15	Extensive reticulonodular shadows, miliary pattern	Findings NR	21^a^	<1	D
8	M	Y	N	N	157	16	37	467	401	17	Left PE	H, ALN	175	9	MDR-TB
9	M	Y	N	Y	117^b^	14	26^c^	387	112	18	Diaphragm pushed up. Other findings NR	H, Sp	13	NR	R
10	F	Y	N	Y	34	14	42	323	451	21	Nodular infiltrates in right lower zone	H, Sp	5	9	R
11	F	Y	N	N	123^b^	42	116	415	326	17	Miliary pattern	Normal	0	NR	R
12	F	Y	N	N	15^b^	6	11	186	78	15	Findings NR	H, ALN	Post-biopsy	1	D
13	F	Y	Y	N	163	21	30^c^	448	278	17	LUZ consolidation, opacities in RUZ, RMZ and LMZ. Hilar lymph nodes	H, ALN, Sp, ascites, (PE, PCE)	39	8	D
14	F	Y	Y	N	20	21	95	2226	1257	26	Fibrosis of right upper lobe, nodular infiltrates in both lower zones	ALN	121^a^	9	R
15	F	Y	Y	N	42^d^	24	33	541	599	24	Small bilateral PE	H	219	>8	XDR-TB
16	F	Y	Y	N	17^d^	65	57	1050	443	21	Right upper lobe infiltrates	ALN, Sp	121^a^	9	R
17	F	Y	Y	N	49	26	28	321	86	11	Bilateral opacification, miliary TB	ALN, Sp	103^a^	6	D
18	F	Y	N	Y	45	6	41	463	1075	20	Reticular infiltrates in RUZ and LUZ	H, ALN	50	6	R
19	M	Y	N	Y	59	32	55	297	321	20	Mediastinal widening, no infiltrates	H, ALN, Sp (PE, PCE)	97	3	D
20	M	Y	N	N	37	13	163	326	460	28	Findings NR	Not done	81^a^	>8	R

*Pt* Patient, *TB* Tuberculosis, *H* hepatomegaly, J Jaundice, *S* Splenomegaly, *CD4* cluster of differentiation 4, *TBil* Total Bilirubin, *ALT* alanine transaminase, *ALP* alkaline phosphatase, *GGT* gamma-glutamyltransferase, *ALB* albumin, *F* Female, *M* Male, *Y* Yes, *N* No, *ALN* Abdominal lymph node/s, ^a^anti-TB therapy interrupted pre-biopsy due to abnormal liver enzymes, *R* Resolved, *D* Died, *RUZ* Right upper zone, *NR* not reported, *PE* Pleural effusion, *MDR-TB* Multidrug-resistant tuberculosis, ^b^on Antiretroviral therapy, ^c^Serology positive for Hepatitis B Surface Antigen, *Sp* Splenic lesions, *LUZ* Left upper zone, *RMZ* Right mid zone, *LMZ* Left mid zone, *PCE* Pericardial effusion, ^d^ART stopped 1–2 months pre-biopsy, *XDR-TB* Extensively drug-resistant tuberculosis

At the time of liver biopsy ten patients had smear-positive pulmonary TB, four smear-negative but culture positive disease and the remaining three were diagnosed by suggestive changes on chest radiograph. Eleven patients (55%) were receiving anti-TB therapy (rifampicin, isoniazid, pyrazinamide and ethambutol) for active pulmonary and/or extra-pulmonary TB disease. Of the three patients who were on anti-TB therapy for extra-pulmonary TB, one was diagnosed by a positive mycobacterial blood culture and the other two on positive culture of pleural effusion and bone marrow respectively (Table 2).

**Table 2:** Microbiology and histology results for 20 patients with hepatic tuberculosis^a^

Pt	Microscopy^b^ and culture of sputum and/or other extrapulmonary site as indicated within ()	Microbiology of liver tissue		Histology of liver tissue^a^
		Culture	Sensitivity	
1	Smear Negative, Culture Positive for MTB	MTB	Pansensitive	AFB seen with granulomatous inflammation.
2	Smear Negative, Culture Positive for MTB	MTB	Pansensitive	AFB seen. Granulomatous inflammation consistent with TB.
3	Smear Positive for MTB	MTB	Pansensitive	AFB seen. Granulomatous inflammation consistent with TB.
4	Smear Positive for MTB	No growth	N/A	AFB seen. Granulomatous inflammation consistent with TB.
5	Smear Positive for MTB	MTB	Pansensitive	AFB seen. Granulomatous inflammation consistent with TB.
6	Smear Negative, Culture Positive for MTB	Not done	N/A	AFB seen with granulomatous inflammation and features of Schistosomiasis.
7	Smear Positive for MTB	MTB	Pansensitive	AFB seen. Granulomatous inflammation consistent with TB.
8	Smear Positive for MTB	No growth	N/A	AFB seen. Granulomatous inflammation consistent with TB.
9	N/A; (bone marrow^c^)	Not done	N/A	AFB seen. Granulomatous inflammation consistent with TB. Hepatitis B positive^d^
10	Smear Positive for MTB	Not done	N/A	AFB seen. Granulomatous inflammation consistent with TB.
11	Smear Positive for MTB	Not done	N/A	AFB seen. Granulomatous inflammation consistent with TB.
12	N/A; (AFB positive on blood culture smear)	AFB grown^e^	Not done	AFB seen with granulomatous inflammation.
13	N/A; (cervical lymph node^c^; ascitic fluid^c^)	No growth	N/A	Granulomatous inflammation consistent with TB.
14	Smear Positive for MTB	No growth	N/A	Granulomatous inflammation consistent with TB.
15	Negative; (pleural effusion^c^)	No growth	N/A	Granulomatous inflammation.
16	N/A	No growth	N/A	Granulomatous inflammation consistent with TB.
17	Smear Positive for MTB	Not done	N/A	AFB seen with granulomatous inflammation.
18	Negative	No growth	N/A	AFB seen. Granulomatous inflammation consistent with TB.
19	Smear Negative, Culture Positive for MTB	No growth	N/A	Features suspicious of Hodgkin’s. Granulomatous inflammation, ZN negative.^f^
20	Smear Positive for MTB	Not done	N/A	Granulomatous inflammation.

^a^Liver histology specimens were stained with Ziehl Neelsen stain to detect acid-fast bacilli. Granulomatous inflammation consistent with TB infers caseating granulomas with foci of activated macrophages (epithelioid cells), rimmed by fibroblasts, lymphocytes, histiocytes and occasional multinucleated giant cells [20]. *Pt* Patient; ^b^Auramine O stain, *MTB* Mycobacterium Tuberculosis; *AFB* acid-fast bacilli, *TB* Tuberculosis, *N/A* Not applicable; ^c^Culture from this biopsy site was positive for MTB; ^d^Immunohistochemistry positive for Hepatitis B virus surface and core antigen; ^e^species not identified; *ZN* Ziehl Neelsen; ^f^histology of bone marrow aspirate showed AFB and granulomatous inflammation consistent with TB

The median duration of anti-TB therapy at liver biopsy was 50 days (range 5–219 days). Six out of twenty patients (30%) had other co-infections: two with Hepatitis B, two with cryptococcal meningitis, one with herpes zoster and one with hepatosplenic schistosomiasis.

### Clinical and radiographic characteristics of hepatic TB

The most frequent clinical finding was hepatomegaly in 17 (85%) patients (Table 1). Other findings included generalized lymphadenopathy (60%), pulmonary crackles (40%), jaundice (35%), and splenomegaly (30%). One patient had ascites.

Chest radiograph reports were available for 17 patients as detailed in Table 1. Abdominal ultrasound reports were available for only 15 patients. Thirteen reported hepatomegaly and/or enlarged intra-abdominal lymph nodes and/or splenic abnormalities, and the remaining two had normal findings.

### Laboratory and histopathology characteristics of hepatic TB

The median CD4 cell count at the time of liver biopsy was 47 cells/μl (inter-quartile range (IQR) 27–107 cells/μl). Liver enzymes at the time of biopsy included elevated alkaline phosphatase (normal range: 42–121 u/l; median 456 u/l, IQR 322–1043 u/l), gamma-glutamyltransferase (normal range: 10–60 u/l; median 422 u/l, IQR 235–736 u/l) and alanine transaminase (normal range: 10–45 u/l; median 46 u/l, IQR 33–95 u/l). All patients had hypoalbuminaemia (normal range: 32–50 g/l; median 17 g/l, IQR 16–21 g/l) and eleven out of twenty patients (55%) had elevated bilirubin levels (normal range: 0–17 μmol/l; median 21 μmol/l, IQR 14–34 μmol/l). The international normalized ratio (INR) was normal in nineteen out of twenty patients (95%) at the time of liver biopsy. The one patient with an abnormal INR had a value of 1.3 (normal range: <1.2).

Liver biopsy tissue samples from 14 patients underwent microscopy and culture (Table 2). All microbiology samples were negative for acid-fast bacilli (AFB) on Auramine stain. Five of the 14 (36%) were culture-positive for TB. All isolates were fully sensitive to isoniazid, rifampicin, streptomycin, kanamycin and ofloxacin. Another patient had mycobacteria isolated from biopsy tissue but this was not worked up further. Histopathologic findings are detailed in Table 2.

### Response to anti-TB therapy and patient outcomes

The changes in serial liver enzyme profiles, as a reflection of response to anti-TB therapy in the first 6 months, were available for twelve out of twenty patients (60%). The liver enzyme fold changes were calculated using the upper limit of normal (ULN) for alkaline phosphatase (ALP) and gamma-glutamyltransferase (GGT), as detailed in Table 3. Eleven out of twelve patients (92%) had reduction with seven out of twelve patients (58%) showing normalization of ALP levels within 6 months of anti-TB therapy.

**Table 3:** Fold change in Alkaline Phosphatase (ALP) and Gamma-Glutamyltransferase (GGT)

	Alkaline Phosphatase			Gamma-Glutamyltransferase		
Patient	Baseline	1 month	6 month	Baseline	1 month	6 month
1	25	11	1.5	25	16	6
2	<ULN	<ULN	<ULN	2	1	<ULN
4	4.5	5	<ULN	7.5	13	<ULN
6	3.5	6.5	<ULN	5.5	17	2
8	2.5	2.5	4	6.5	4	1
9	2	1	<ULN	<ULN	<ULN	<ULN
10	1.5	2.5	<ULN	6.5	9.5	6.5
13	2	4.5	1.5	2.5	5.5	1
14	17.5	7	5.5	20	9.5	16.5
15	3.5	5.5	<ULN	9	10	2.5
16	<ULN	3	<ULN	<ULN	3	<ULN
17	9.5	8.5	3.5	19.5	13	5.5

Fold change is calculated by dividing patients’ value by upper limit of normal (ULN). Alkaline phosphatase ULN = 121 U/L, Gamma Glutamyltransferase ULN = 60 U/L

The duration of anti-TB therapy was clearly documented in seventeen out of twenty patients (85%). Median duration of anti-TB therapy was 9 months with a range of 1 to 13 months. Twelve out of twenty patients (60%) were successfully treated with anti-TB therapy. Two out of twenty patients (10%) developed drug resistant TB, one at 6 months and the other at 11 months after liver biopsy. At the time of hepatic TB diagnosis both had drug susceptible TB based on isolates from extra-hepatic sites. Both patients died at least one year after liver biopsy and within 36 months of the diagnosis of drug resistant TB. The mortality in our study at one year post-biopsy was 30% and median time to death was 4 months (range <1 to 8 months).

## Discussion

We characterized the clinical and histopathologic features, as well as the response to treatment of hepatic TB in HIV co-infected patients. In our case series, hepatic TB occurred mostly in persons with severe immunosuppression [14, 15, 22]. The age distribution in this series closely matched that of HIV infection, due to the close association between extra-pulmonary TB and HIV [8, 22]. The clinical features of hepatic TB in our study were similar to those reported from HIV-uninfected case series [4, 7, 9]. A notable exception was that two thirds of our patients had generalized lymphadenopathy, which could be a manifestation of TB disease or reactive adenopathy characteristic of HIV infection [23].

Consistent with the findings of others, hepatic TB caused a disproportionate elevation of the biliary canalicular enzymes, likely reflecting an infiltrative pattern by granulomatous inflammation [7–9, 14, 15, 24, 25]. The low serum albumin should not be interpreted as a marker of hepatocellular synthetic dysfunction since it is influenced by multiple factors such as malnutrition, chronic infection, chronic inflammation and HIV-associated nephropathy [7, 8, 14, 26]. Coagulation studies are a more reliable marker of hepatic function, however these were normal in our case series which may imply that granulomatous inflammation does not interfere with hepatocellular function [24].

Eighty five percent (85%) of our case series had features of pulmonary tuberculosis suggesting that hepatic involvement was probably secondary to dissemination from the lung. However only two chest radiographs showed the typical pattern of TB lung involvement, a finding consistent with the severity of the underlying immunodeficiency [1, 27]. Microbiology of liver tissue demonstrated poor sensitivity in confirming a diagnosis of hepatic TB [15, 22]. Prior exposure to anti-TB therapy might have played a role, and the unreliability of microbiology was well recognized in the pre-HIV era [4, 7, 9, 11].

The histologic findings of hepatic TB include epithelioid granuloma, often with caseation and non-specific reactive hepatitis [28]. In advanced HIV disease, granuloma formation in response to mycobacterial infection is poor and may be associated with increased bacillary presence [15, 25, 29]. Hepatic TB granuloma formation has been well described in severe HIV disease upon commencement of ART, as a manifestation of IRIS [22]. In this study, the histologic findings showed varying degrees of granulomatous inflammation with no relationship between the CD4 cell count and the presence or absence of granulomatous inflammation. Most of the patients in this study were not receiving ART, suggesting that a granulomatous response was possible despite a poorly functioning immune system. Although a hepatic granulomatous response may be associated with IRIS we were not able to draw any conclusions from our predominantly ART-naïve case series [22].

Due to concerns of exposing patients with unexplained liver enzyme abnormalities to potentially hepatotoxic drugs, 15 of the 20 (75%) patients were not commenced on ART despite clear indication and a further 2 (10%) on ART had their ART discontinued. Such treatment delays or interruptions could result in serious consequences. Thus it is important to recognize that the described liver enzyme abnormalities, in the context of TB at other sites, is possibly indicative of hepatic TB and should not unnecessarily delay ART.

Optimal duration of anti-TB therapy for hepatic involvement remains unclear [15] and changes in liver enzyme abnormalities may be used to monitor response to anti-TB therapy. Based on trends of liver enzyme changes available for twelve patients, liver enzyme levels should normalize in most patients within six months of anti-TB therapy. However, it is unclear if one should use normalization of the liver enzyme abnormalities to determine when to discontinue treatment. Literature shows that there may be up to 30% variability of liver enzymes due to patient factors [30] and in some of our patients, enzyme abnormalities resolved several months after cessation of anti-TB therapy.

The treatment success rate in our case series was lower than that reported for sub-Saharan Africa [2]. Advanced immunosuppression may have contributed to the high mortality rate, consistent with other TB studies [3].

Our study does have some limitations. Patients described in our study were all managed in an Infectious Diseases Unit at King Edward VIII hospital, which is a tertiary level institute. Hence, it is likely that many patients with possible hepatic TB were managed empirically, based on evidence of TB in other organ systems, at the peripheral health care facility. Therefore, this case series represents a more select group with a particularly high suspicion of hepatic TB. Furthermore, patterns of liver enzyme abnormalities might have biased patient selection for liver biopsy.

Like any retrospective case series study design, we cannot draw any conclusions regarding incidence or prevalence of disease [31]. However, our observations highlight when one should be suspicious of hepatic TB and offers guidance on monitoring response of hepatic TB to anti-TB therapy.

## Conclusion

Clinicians should maintain a high index of suspicion for hepatic TB, especially among HIV-infected patients with elevated liver enzymes and evidence of TB in other organ systems. The presence of unexplained hepatomegaly and preserved liver synthetic function are additional supportive features. Such enzyme abnormalities should not delay the initiation of ART. Granulomatous reactions are a common histopathologic finding even in advanced HIV disease and microbiology of liver tissue has a role in the diagnosis of hepatic TB. Evolution of liver enzyme abnormalities in response to anti-TB therapy might serve as a useful monitoring tool but the decision to discontinue anti-TB therapy should not depend on normalization of liver enzymes since this may take several months to occur.

## Abbreviations

AFB: Acid-fast bacilli
ALP: Alkaline phosphatase
ART: Anti-retroviral therapy
CD4: Cluster of differentiation 4
GGT: Gamma-glutamyltransferase
HIV: Human immunodeficiency virus
IDC: Infectious diseases clinic
INR: International normalized ratio
IQR: Inter-quartile range
IRIS: Immune reconstitution inflammatory syndrome
REDCap: Research electronic data capture
TB: Mycobacterium tuberculosis
ULN: Upper limit of normal
